# Gender-related differences in physiologic color space: a functional transcranial Doppler (*f*TCD) study

**DOI:** 10.1186/2040-7378-3-1

**Published:** 2011-02-10

**Authors:** Philip C Njemanze

**Affiliations:** 1 Chidicon Medical Center, Owerri, Imo State, Nigeria

## Abstract

Simultaneous color contrast and color constancy are memory processes associated with color vision, however, the gender-related differences of 'physiologic color space' remains unknown. Color processing was studied in 16 (8 men and 8 women) right-handed healthy subjects using functional transcranial Doppler (*f*TCD) technique. Mean flow velocity (MFV) was recorded in both right (RMCA) and left (LMCA) middle cerebral arteries in dark and white light conditions, and during color (blue and yellow) stimulations. The data was plotted in a 3D quadratic curve fit to derive a 'physiologic color space' showing the effects of luminance and chromatic contrasts. In men, wavelength-differencing of opponent pairs (yellow-blue) was adjudged by changes in the RMCA MFV for Yellow plotted on the *Y*-axis, and the RMCA MFV for Blue plotted on the *X*-axis. In women, frequency-differencing for opponent pairs (blue-yellow) was adjudged by changes in the LMCA MFV for Yellow plotted on the *Y*-axis, and the LMCA MFV for Blue plotted on the *X*-axis. The luminance effect on the LMCA MFV in response to white light with the highest luminous flux, was plotted on the (*Z *- axis), in both men and women. The 3D-color space for women was a mirror-image of that for men, and showed enhanced color constancy. The exponential function model was applied to the data in men, while the logarithmic function model was applied to the data in women. Color space determination may be useful in the study of color memory, adaptive neuroplasticity, cognitive impairment in stroke and neurodegenerative diseases.

## Introduction

Simultaneous color contrast and color constancy are memory processes associated with color vision [1-8], however, the gender-related differences of 'physiologic color space' remains unknown. Simultaneous color contrast is the phenomenon that surrounding colors profoundly influence perceived color [1,9]. The mechanism for simultaneous color contrast involves wavelength-differencing [10]. However, recently, it was suggested that, simultaneous color contrast involved wavelength-differencing in men [11], while in women, implicated frequency-differencing [12]. The latter study implemented functional transcranial Doppler spectroscopy (*f*TCDS) technique, which demonstrated the process of wavelength-encoding, whereby the effects of longer wavelength color (Yellow) was accentuated over shorter wavelength color (Blue). The converse process of energy-encoding accentuated the effects of higher frequency color (Blue), over lower frequency color (Yellow). Wavelength-differencing involves wavelength-encoding main effect at subcortical peaks, and energy-encoding effects at cortical peaks [11]. On the other hand, frequency-differencing involved energy-encoding main effect at cortical and subcortical peaks [12]. Beyond the physiologic evidence, genetic studies suggest gender-related differences in color vision in primates. Whereas both trichromatic and dichromatic color vision occurs among female monkeys, males appear exclusively dichromatic [13]. It was proposed that unlike humans, squirrel monkeys have only a single photopigment locus on the X chromosome [13]. The presumed genetic and psychophysiologic effects of the physical qualities of light underlie the proposal for light hypothesis of cerebral asymmetry [12].

Color constancy relates to the invariance of hue, or perceived color of a surface under variation in the spectral content of illumination [3,14,15]. More specifically, color constancy refers to the unchanging nature of the perceived color of an object despite considerable variation in the wavelength composition of the light illuminating it [16]. The processes underlying color constancy and simultaneous color contrast are functionally integrated within the color space in the brain. However, the mechanisms implicated in the integration of chromatic and achromatic contrasts remains unclear.

It has been proposed that there may exist a color space comprising chromatic axes (red-green and blue-yellow) and achromatic (black-white) luminance axis [8]. However, obtaining non-invasive physiologic measurements at the neural substrates to characterize the 'color space' in humans has not been feasible until now. The main neural substrates for color processing have been identified within the visual pathways and extrastriate cortex 'color centres' [17], which lie within the distribution territories of the posterior cerebral arteries (PCAs) [18-20] and middle cerebral arteries (MCAs) [18,20,21]. The superior parietal aspects of the optic radiation receive blood mainly from the MCA, whereas the inferior aspects are supplied by the PCAs [22]. The macular cortical region receives blood supply, from both the calcarine artery (a branch of the internal occipital artery of the PCA distribution), and branches of the MCA, which explains macular sparing with PCA occlusion [18]. It is therefore plausible to determine the 'color space' in the primary visual cortex and extrastriate areas using measurements of changes in blood flow velocity in the MCAs during color stimulation [21]. Prior studies in our laboratory using non-invasive transcranial Doppler measurement of mean flow velocity (MFV) in the MCAs, have examined changes in color space in men during head-down rest, and demonstrated neuroplasticity over 25 hours of recordings [11], or more, if testing was continued. These findings have been related to formation of the phenomena of long-term potentiation (LTP) and long-term depression (LTD), implicating role for NMDA receptors [11].

The phenomena of LTP [23] and LTD [24], have been applied to explain potential mechanisms of memory, primarily because they exhibit numerous properties expected of a synaptic associative memory mechanism, such as rapid induction, synapse specificity, associative interactions, persistence, and dependence on correlated synaptic activity. Given these important features, LTP and LTD remains only models of the synaptic and cellular events that may underlie memory formation. Recently, it was demonstrated using functional transcranial Doppler spectroscopy (*f*TCDS) technique, that color processing occurred within cortico-subcortical circuits [11,12]. It was demonstrated that in men, wavelength-differencing of Yellow/Blue pairs occurred within the right hemisphere by processes of cortical long-term depression (CLTD) and subcortical long-term potentiation (SLTP). On the other hand, in women, frequency-differencing of Blue/Yellow pairs occurred within the left hemisphere by processes of cortical long-term potentiation (CLTP) and subcortical long-term depression (SLTD) [11,12]. In both genders, there was luminance effect in the left hemisphere, while in men it was along an axis orthogonal to chromatic effect, in women it was along a parallel axis [11,12]. It therefore, may be possible to describe mechanisms for color memory formation using *f*TCD and *f*TCDS techniques.

In the present work, it was hypothesized that, the brain functionally integrates within a three-dimensional physiologic 'color space', luminance effect in the left hemisphere with wavelength-differencing activity in the right hemisphere in men, but with frequency-differencing activity in the left hemisphere in women. This functional integration in color space could be mathematically modelled and could have potential applications in the study of color memory, adaptive neuroplasticity in stroke management and rehabilitation, as well as in neurodegenerative diseases.

## Methods

The study included 8 men of mean ± SD age of 24.6 ± 2 years, and 8 women of mean ± SD age of 24 ± 2 years, all were right handed on hand preference questionnaire [25]. Testing of visual acuity by Snellen chart, color vision by Ishihara pseudoisochromatic plates and color recognition were normal [26]. There were no abnormalities uncovered during routine evaluation of the cardiovascular, neurologic and respiratory systems. All were urged to maintain the usual restrictions for cognitive studies [27]. All subjects gave written informed consent according to the Declaration of Helsinki; and the Institutional Ethics Board approved the study protocol. The experimental procedure including TCD scanning have been used for cognitive studies and described in detail elsewhere [11,21]. In summary, TCD studies were performed using two 2 MHz probes of a bilateral simultaneous TCD instrument (Multi-Dop T, DWL, Singen, Germany), with sample volume placed in the RMCA and LMCA main stems at a depth of 50 mm. The measurements in the MCAs which supply about 80% of brain blood flow [28], would provide a more global characterization of the color space than those from the PCAs. The recordings were performed with the subject lying supine, with head and trunk elevated at 30 degrees.

### Tasks

The tasks were designed in our laboratory and the detailed rationale described elsewhere [21]. These tasks have proven to be consistent and reliable with TCD ultrasonography in prior studies [11,21]. In summary, specially adapted 3D-viewing device (Viewmaster, Portland, OR) was painted inside with black paint. The right aperture of the device was closed to light, but the left aperture was open, to be backlit from white light reflected from a remotely placed light source. In other words, there was left eye monocular vision, with light path from the left visual hemifield reaching the right side of the left eye retina, and crossing at chiasm to project contralaterally to the right visual cortex, while the light path from the right visual hemifield reaching the left side of the left eye retina, project ipsilaterally to the left visual cortex. The rationale relates to the fact that, in primates including humans, there is virtually total binocular vision; the left half of each retina project to the left visual cortex and the right half of each retina project to the right visual cortex. This means that the right visual cortex receives all its input from the left visual field and the left visual cortex receives all its inputs from the right visual field [29]. Color processing cells, receiving inputs from only one eye are grouped together within the same area of the striate cortex, extending from the upper to the lower cortical layers, and are referred to as ocular dominance column (blobs) [15,30,31]. On the other hand, those receiving inputs from both eyes are called hypercolumn [30,32]. During binocular vision, there is binocular interaction due to stereopsis, the perception of depth [15]. It is inappropriate to mix the inputs, from both retinas in a single neuron, before the information of color vision has been extracted [15].

The light source was projected onto a white surface flat screen, placed 125 cm from the lamp. The screen was positioned 80 cm from the nose ridge of the subject. The light source was a tungsten coil filament, of a general service lamp ran at a constant 24 V and 200 W, with a color temperature of about 2980 K and approximately 20 lumens/watt.

Color stimulation was performed using optical homogenous filters placed on the reel of the Viewmaster, in the light path. Kodak Wratten filters: Deep Blue (No. 47B) with short dominant wavelength (λ) of ***S***_λ _= 452.7 nm; and Deep Yellow (No. 12) with medium dominant wavelength (λ) of ***M***_λ _= 510.7 nm were used. The manufacturer's manual provides the excitation purity and luminous transmittance for each filter [33].

### Visual Stimulation and Recording

Measurements in dark condition were considered as light absent condition, as well as including background effects of scotopic vision [19,34]. It is known that black may evoke a color experience [34]. A continuous train of velocity waveform envelopes, was recorded for 60-s simultaneously, for the RMCA and LMCA, respectively, for each stimulus condition. The baseline condition was dark resting state, with the subject mute, still, and attention focused and eyes fixed at the centre within the dark visual field, with no mental or manual tasks to perform. An observer monitored the subject for movement artefacts, which were marked and removed from recordings. During color stimulation the condition was identical to that of baseline, except for use of slides that were Blue, and Yellow, respectively. Measurements with the left aperture on the reel open to white light reflected from the remote light source was considered as White light stimulation. Velocity waveform envelopes for the relevant 60-s intervals were first averaged in 10-s segments, to produce six values for each measurement condition. The resulting values were used for further calculations. Electrophysiological monitoring of pulse and respiration rates were recorded. Self-perceived anxiety levels were monitored with a state-trait anxiety inventory (STAI) in pre-test and post test conditions [35,36].

### Three Dimensional Color Space

It could be presumed that opponency was accomplished across two orthogonal coordinates of blue and yellow, respectively [11]. In men, wavelength-differencing activity was captured by changes in the RMCA MFV for Yellow plotted on the *Y*-axis, and the RMCA MFV for Blue plotted on the *X*-axis [11]. In women, frequency-differencing [12] was captured by changes in the LMCA MFV for Yellow plotted on the *Y*-axis, and the LMCA MFV for Blue plotted on the *X*-axis. The luminance effect on the LMCA MFV in response to White light with the highest luminous flux, was plotted on the (*Z *- axis), in both men and women [11]. The 3D-color space plots were reconstructed using the quadratic function [11], fitted by the procedure that has the general form:

$$Z={\text{a}}_{\text{o}}+{\text{a}}_{\text{1}}X+{\text{a}}_{\text{2}}\text{Y}+{\text{a}}_{\text{3}}X^{\text{2}}+{\text{a}}_{\text{4}}Y^{\text{2}}+{\text{a}}_{\text{5}}XY.\;$$

The 3D-surface plot offers a flexible tool, for approximating the relationships between observed variables. The quadratic surface plot does not flex to accommodate local variations in data. When the overall pattern of changes in MFV dataset follows some segment of the quadratic surface, then a good fit could be achieved. The goodness of fit was examined, by displaying the raw data spikes on the surface, and the influence of outliers was assessed. The MFV gradient produced color scale sequence ranges (printed in color online), from minimum (green) to maximum (red) of *Z*-values of luminance effect. The color sequence ranges were used to adjudge the level of luminance effect required for wavelength-differencing activity in men, and frequency-differencing activity, in women.

### Exponential Function Model

In men, the 3D-graph was examined to uncover the relationship between luminance effects on the LMCA MFV for White light (*Z*-axis) and wavelength-differencing on the RMCA MFV for Yellow (*Y*-axis). The observation suggested that, the data could be fitted to an exponential function model. The LMCA MFV for White light (*Y*-axis) and RMCA MFV for Yellow (*X*-axis) were fitted to an exponential function of a 2D graph, given the following:

The exponential function with positive base *b *>1 is the function

$$y\;=\;b^{x}.$$

**Figure 1:**
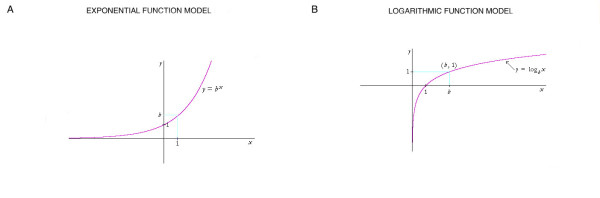
**A shows the exponential function curve used for any base *b*, and horizontal asymptote: Figure 1B shows the logarithmic function curve used for any base *b*, and vertical asymptote**.

It is defined for every real number *x*. The graph is shown in Figure 1A for any base *b*.

There are two important things to note:

• The *y*-intercept is at (0, 1). For, *b*^0 ^= 1.

• The negative *x*-axis is a horizontal asymptote. For, when *x *is a large negative number -- e.g. *b*^-10,000 ^- then *y *is a very small positive number.

### Logarithmic Function Model

In women, the 3D-graph was examined to uncover the relationship between luminance effect on the LMCA MFV for White light (*Z*-axis) and frequency-differencing on the LMCA MFV for Blue (*X*-axis). The observation suggested that, the data could be fitted to a logarithmic function. The LMCA MFV for White light (*Y*-axis) and LMCA MFV for Blue (*X*-axis) were fitted to a logarithmic function of a 2D graph, given the following:

The logarithmic function with base *b *is the function

$$y\;=\;{\text{log}}_{b}x.$$

*b *is normally a number greater than 1 (although it need only be greater than 0 and not equal to 1). The function is defined for all *x *>0. Figure 1B shows the graph for any base *b*. The negative *y*-axis is a vertical asymptote.

### Inverse relations of Exponential and Logarithmic Functions

The inverse of any exponential function is a logarithmic function. For, in any base *b*:

$$i)\;\;\;b^{\text{log}}{{}_{b}}^{x}=x,$$

and

$$ii)\;\;\;{\text{log}}_{b}b^{x}=x.$$

Rule *i*) embodies the definition of a logarithm: log_*b*_*x *is the *exponent *to which *b *must be raised to produce *x*.

Now, let

$$\lambda (x)=b^{x}\;\text{and}\;f(x)={\text{log}}_{b}x.$$

Then Rule *i*) is λ(*f*(*x*)) = *x*.

And Rule *ii*) is *f*(λ(*x*)) = *x*.

These rules satisfy the definition of a pair of inverse functions. Therefore for any base *b*, the functions

$$\lambda (x)=b^{x}\;\text{and}\;f(x)={\text{log}}_{b}x$$

are inverses.

This defines the well established relationship between wavelength (λ) and frequency (*f*), respectively, that is given by:

λ=c/f.

where *c *is the speed of light in a vacuum and remains constant at 3.00 × 10^8 ^m/s.

## Results

**Figure 2:**
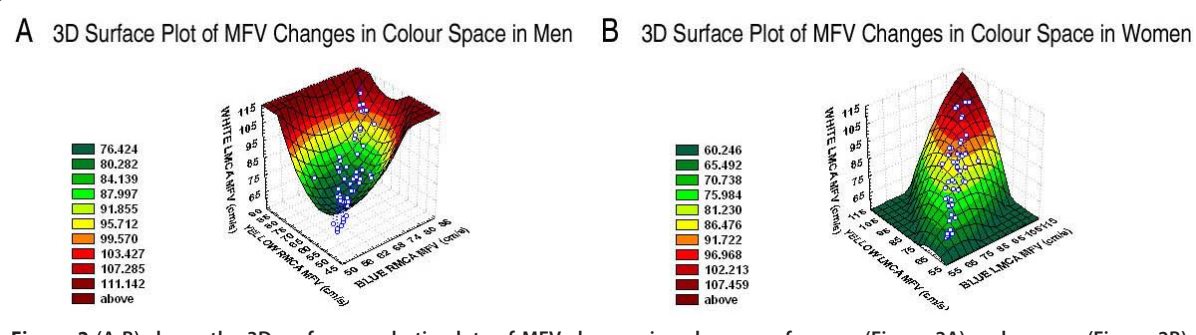
**(A-B) shows the 3D surface quadratic plots of MFV changes in color space for men (Figure 2A) and women (Figure 2B), respectively**.

**Figure 3:**
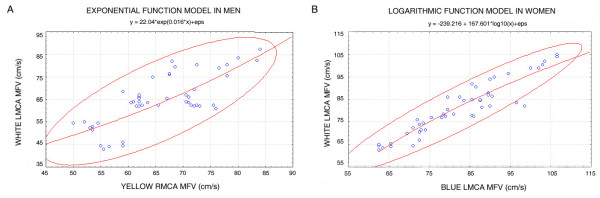
**(A-B) shows the exponential function model fitted to the data in men (Figure 3A), and the logarithmic function model fitted to the data in women (Figure 3B)**. All subjects were plotted with the 95% confidence band shown.

Table 1 shows the Mean ± SE of MFV obtained during visual stimulation in men and women, respectively. Figure 2(A-B) shows the 3D surface quadratic plots of MFV changes in color space for men (Figure 2A) and women (Figure 2B), respectively. In men, the 3D-surface quadratic plot (Figure 2A) was funnel shaped, indicating overall that, wavelength-differencing activity was narrowed at low luminance effect, but broadened with increasing luminance effect. In men, there was an exponential relationship between right hemisphere wavelength-differencing and contralateral left hemisphere luminance effect, as demonstrated in the 2D graph (Figure 3A), showing all subjects within the 95% confidence band. The exponential function model in men (Figure 3A) indicated that brain functional integration of luminance effects and wavelength-differencing activities was maintained within a 'narrow physiologic range' of MFV of 50 to 85 cm/s in the RMCA and LMCA.

**Table 1:** Mean ± SE of MFV (cm/s) during Visual Stimulations in Men and Women.

Dark		Light		Blue		Yellow		
RMCA	LMCA	RMCA	LMCA	RMCA	LMCA	RMCA	LMCA	Men
	
64 ± 1.26	63.9 ± 1.8	65.7 ± 1.2	64.2 ± 1.67	66.8 ± 1.28	65.6 ± 1.8	65.6 ± 1.26	64.4 ± 1.7	

RMCA	LMCA	RMCA	LMCA	RMCA	LMCA	RMCA	LMCA	Women
	
81.6 ± 2	80.7 ± 1.8	82.3 ± 2	81.26 ± 1.7	83.8 ± 1.9	81.6 ± 1.77	82.57 ± 1.86	81.3 ± 1.6	

Conversely, the 3D-surface quadratic plot in women was the mirror-image of that observed in men, showing a closed 'cone shape' with a widespread base (Figure 2B). The base of the cone shape suggests that at very low luminance effect on LMCA MFV, frequency-differencing activity occurred over a very wide range. However, with increasing luminance effect, frequency-differencing activity narrowed. In women, there was a logarithmic relationship between ipsilateral left hemisphere frequency-differencing and luminance effect, as demonstrated in the 2D graph of logarithmic function (Figure 3B), showing all subjects but two, within the 95% confidence band. The logarithmic function model in women (Figure 3B) indicates that, brain functional integration of luminance effect and frequency-differencing activities was maintained within a 'narrow physiologic range' of MFV of 60 to 106 cm/s in the LMCA, somewhat wider range and shifted upwards than that for men.

## Discussion

This is the first published report in literature, using non-invasive *f*TCD to determine gender-related differences in 'physiologic color space'. Overall, the gender-related differences could be summarized as follows: (1) In men, wavelength-differencing activity was enhanced at high luminance effect, conversely, in women, frequency-differencing activity was enhanced at low luminance effect. (2) In men, luminance effect varied exponentially with wavelength-differencing activity, while in women, luminance effect varied logarithmically with frequency-differencing activity. (3) The physiologic range of MFV in the MCA territories for the relationship between luminance effect and wavelength-differencing activity in men, was narrower than that for frequency-differencing activity in women.

It has been suggested that the right hemisphere was implicated in color processing [11,12,19,37,38] and the left hemisphere in color memory [39,40]. Functional magnetic resonance studies demonstrated that memory for colors activated the same cortical regions associated with color perception in the left fusiform gyrus [39,40]. This has led some investigators to suggest that, color memory is a constructive process reflecting the synthesis of features each of which are processed in the different cortical regions [40-43]. The functional integration of color processing and luminance effect could provide a model for the study of the functional integration of color processing and color memory within the color space. Given what is known thus far, it could be proposed that in men, wavelength-differencing within the right hemisphere by processes of cortical long-term depression (CLTD) and subcortical long-term potentiation (SLTP) [12], occurred simultaneously with contralateral cortical short-term depression (CSTD) and subcortical short-term potentiation (SSTP) in the left hemisphere, marked by exponential decaying increase in synaptic strength that appears to involve NMDA receptors as well [44,45], but decays after reaching the asymptotic levels of MFV (Figure 3A). Thus, it could be postulated that in men, in the contralateral left hemisphere, memory activation implicated 'exponential expansion' by CSTD and SSTP processes. On the other hand, in women, frequency-differencing within the left hemisphere occurred by processes of cortical long-term potentiation (CLTP) and subcortical long-term depression (SLTD) [12], concurrently, with ipsilateral cortical short-term potentiation (CSTP) and subcortical short-term depression (SSTD) in the left hemisphere [12,44,45], characterized by logarithmic decaying decrease in synaptic strength that may also involve NMDA receptors, but decays after reaching the asymptotic levels of MFV (Figure 3B). Thus, in women, in the ipsilateral left hemisphere memory activation involved 'logarithmic compression' by CSTP and SSTD processes. Analogous synaptic and cellular activities have been observed in animal experiments [44,45].

The holistic-synthetic versus analytic gender-related cognitive styles have been proposed for hemispheric asymmetry for facial processing [46-48], and general intelligence [49-51]. The synthetic versus analytic divide may underscore that, color processing did not impose any new constraints on the brain other than that implemented for other stimuli [46-51]. It could be presumed that, the constructive synthetic processing of color memory in men, implicated synaptic and cellular activities that accomplished exponential expansion in memory in the contralateral left hemisphere. On the other hand, in women, the synaptic and cellular activities accomplished logarithmic compression in memory in the ipsilateral left hemisphere. Given that the constructive synthetic memory processing in men, would require encoding and retrieval processes through trans-callosal pathways from the right hemisphere to the contralateral left hemisphere, both temporal and quantitative color memory formation would be expected to be less efficient in comparison to ipsilateral left hemisphere analytic processing in women. Furthermore, the ipsilateral left hemisphere processing of frequency-differencing and luminance effect, in women, would allow greater flexibility for color constancy in women than in men [52]; as well as the co-localization, may enhance color memory and naming capabilities in women [53-58]. Some have identified that, women access a larger repertoire of words to describe colors across ages, languages and cultures, and are better at color matching from memory [53-58].

The existence of wavelength-differencing in men and frequency differencing in women, which implement inverse exponential and logarithmic functions respectively, may be related to the dual nature of light as a wave and as particulate energy referred to as quanta [11,12]. The reason for the male preference for right hemisphere wavelength-differencing, and female preference for left hemisphere frequency-differencing remains unknown. However, according to the light hypothesis for cerebral asymmetry, which posits that, the phenotypic neuroadaptation to environmental physical constraints of light as a wave and as quanta energy led to phenotypic evolution, and genetic variation of X-Y gene pairs that determine hemispheric asymmetry [12]. Hence, the evolutionary trend is towards optimization of perception of the 'whole' environment by functional coupling of the genes for complementarity of both hemispheres within self, and between both genders [12]. The wavelength-differencing in men and frequency-differencing in women may be physical expressions with neurobehavioral and psychophysiologic correlates for cognitive styles.

The results support the notion of adaptive neuroplasticity within the visual system. The functional modulation by luminance effect of wavelength-differencing in men, and frequency-differencing in women, is a manifestation of adaptive neuroplasticity within the visual system. Preliminary work in healthy men, during head-down rest showed adaptive changes in 3D color space in different head positions and return to normal conditions [11]. Such adaptive neuroplasticity would be expected to be constrained by disease processes. The present work offers a potential approach to non-invasive assessment of adaptive neuroplasticity within a functionally specific region of the brain with simple selective visual color stimuli, which lacks major confounding effects of other modalities. It is plausible that changes in color space may correlate with early signs of cognitive impairment. However, it remains to be demonstrated in disease conditions such as stroke and neurodegenerative diseases.

Furthermore, the results exemplify nonlinearity in the human visual system. The nonlinearity response modelled by exponential and logarithmic functions, respectively, showed a measure of semi-linearity within the physiologic range of MFV, but not beyond these limits. This may suggest that, there exists a narrow physiologic range of cerebral blood flow velocity, within which there was regulation of visual processing, in much the same manner as cerebral autoregulation, during fluctuations of blood pressure. It could be presumed that, for cognitive performance not to be compromised, there exits a narrow physiologic range for fluctuation of cerebral blood flow velocity in the MCAs. It is plausible that, this narrow physiologic range within the modelled exponential and logarithmic curves may provide a potential estimation of 'cognitive autoregulation'. In men, the exponential function yielded a negative *X*-axis horizontal asymptote (Figure 1A and Figure 3A). The latter may suggest that, normal wavelength-differencing along the *X*-axis (RMCA MFV for Yellow) would seize to occur at a critically lower limit of luminance effect on LMCA MFV (*Y*-axis). On the other hand, in women, the logarithmic function yielded a negative *Y*-axis (LMCA MFV for White light) vertical asymptote (Figure 1B and Figure 3B). The latter may suggest that frequency-differencing along the *X*-axis (LMCA MFV for Blue) may occur with absent luminance effect, that is, during scotopic vision in women. Moreover, it has been suggested that women have high scotopic sensitivity in the right hemisphere and may contribute to this improved capability [19,46]. However, this remains to be tested using a variety of visual stimuli. Pathologic conditions such as stroke and neurodegenerative diseases, with resulting cerebral hypoperfusion could condition a shift of the curves to the right, downwards and narrowing of the range. Future studies would explore these changes in correlation with early signs of cognitive impairment. It is therefore plausible that, progressive monitoring of the curve shift overtime may be of diagnostic value, and may well be used in monitoring stroke rehabilitation.

The existence of a physiologic color space in the human brain has long been presumed, however, direct physiologic measurements to compute a color space have not been possible until now. Conventional approaches assess color space by perceptual color representation in a coordinate system [59]. The present work demonstrates that a color space could be constructed from responses to opponent colors (blue-yellow) and luminance effects, eliminating the need for multiplicity of colors used in conventional systems that compound assessment of perceptive responses. Conventional color image processing algorithms do not consider nonlinearity of human color vision and the limitations of the human vision to discriminate between two similar colors and the maximum number of colors human vision can respond to. It is plausible that, the physiologic color space could be transformed to perceptual correlates that comprise a variety of other colors within a composite color space. The resulting perceptual color space would be of immense interest to color vision researchers, seeking ways to improve visual graphics on computers.

The functional integration within the color space was useful for understanding mechanisms implicated in simultaneous color contrast, color constancy, and formation of color memory, as constructive synthetic in men, but analytic in women. In conclusion, the paradox of color perception is that men and women see and remember it differently; however, the essence of this sex dimorphism is complementarity of the genders, aimed at enhancing our collective experience of the physical World around us.

## Disclosures

The author declares that they have no competing interests.
